# Combating the COVID-19 Pandemic: Experiences of the First Wave From Nepal

**DOI:** 10.3389/fpubh.2021.613402

**Published:** 2021-07-12

**Authors:** Buddha Bahadur Basnet, Kiran Bishwakarma, Ramesh Raj Pant, Santosh Dhakal, Nashib Pandey, Dhruba Gautam, Archana Ghimire, Til Bahadur Basnet

**Affiliations:** ^1^Faculty of Science, Nepal Academy of Science and Technology, Lalitpur, Nepal; ^2^Nepal Environment and Development Consultant Pvt. Ltd., Kathmandu, Nepal; ^3^Central Department of Environmental Science, Institute of Science and Technology, Tribhuvan University, Kathmandu, Nepal; ^4^Nepal Development Society, Bharatpur, Nepal; ^5^Kantipur Dental College Teaching Hospital and Research Center, Kathmandu University, Kathmandu, Nepal; ^6^National Disaster Risk Reduction Centre, Kathmandu, Nepal; ^7^Little Buddha College of Health Sciences, Kathmandu, Nepal

**Keywords:** COVID-19, pandemic, preparedness, response, spatial distribution, public health, Nepal

## Abstract

Unprecedented and unforeseen highly infectious Coronavirus Disease 2019 (COVID-19) has become a significant public health concern for most of the countries worldwide, including Nepal, and it is spreading rapidly. Undoubtedly, every nation has taken maximum initiative measures to break the transmission chain of the virus. This review presents a retrospective analysis of the COVID-19 pandemic in Nepal, analyzing the actions taken by the Government of Nepal (GoN) to inform future decisions. Data used in this article were extracted from relevant reports and websites of the Ministry of Health and Population (MoHP) of Nepal and the WHO. As of January 22, 2021, the highest numbers of cases were reported in the megacity of the hilly region, Kathmandu district (population = 1,744,240), and Bagmati province. The cured and death rates of the disease among the tested population are ~98.00 and ~0.74%, respectively. Higher numbers of infected cases were observed in the age group 21–30, with an overall male to female death ratio of 2.33. With suggestions and recommendations from high-level coordination committees and experts, GoN has enacted several measures: promoting universal personal protection, physical distancing, localized lockdowns, travel restrictions, isolation, and selective quarantine. In addition, GoN formulated and distributed several guidelines/protocols for managing COVID-19 patients and vaccination programs. Despite robust preventive efforts by GoN, pandemic scenario in Nepal is, yet, to be controlled completely. This review could be helpful for the current and future effective outbreak preparedness, responses, and management of the pandemic situations and prepare necessary strategies, especially in countries with similar socio-cultural and economic status.

## Introduction

The unanticipated outbreak of the novel coronavirus was first reported in Wuhan, China, in December 2019; it transmits from human to human *via* droplets and aerosol (1). The WHO declared Coronavirus Disease 2019 (COVID-19) as a Public Health Emergency of International Concern (PHEIC) on January 30, 2020, and a pandemic on March 11, 2020 (2). As a result, countries worldwide adopted various mitigative measures (3, 4) and eradication strategies (5), aiming to reduce potentially enormous damage and reach zero cases, respectively. However, significant gaps in advance preparedness and the implementation of response plans resulted in the rapid spread of severe acute respiratory syndrome coronavirus-2 (SARS-CoV-2) globally with 219 nations reporting it as of January 22, 2021^1^ (6).

The Federal Democratic Republic of Nepal is a landlocked country in South Asia bordered by India in the south, east, and west, and China in the north. Its population, gross domestic product (GDP), and human development index (HDI) are 29.24 million^2^, 30.64 billion^3^, and 0.579^4^, respectively. The constitution of Nepal (2015) consists of a three-tier (federal, province, and local) governmental system. Each tier has the constitutional power to enact laws and mobilize its resources. In Nepal, the first case of COVID-19 was reported on January 23, 2020, in a 32-year-old Nepalese man who returned from Wuhan, China. Two months after the first case, the second case was diagnosed through domestic testing on March 23 in a returnee from France (7). Subsequently, the Government of Nepal (GoN) imposed early interventions approved by the WHO, including a travel ban and the Indo-Nepal and China-Nepal borders closure^5^ (8) to delay the possible onset of the detrimental effects of the outbreak across the country.

This review presents a 1-year (up to January 22, 2021) scenario of COVID-19 in Nepal, reviews the strategies employed by the GoN to control COVID-19, and provides suggestions for the prevention and control of current and future pandemics. Federal, provincial, and district-level daily cases of COVID-19 [confirmed by real-time PCR (qRT-PCR), cured, and death] in Nepal from January 23, 2020, to January 22, 2021, were obtained from the Ministry of Health and Population (MoHP), GoN^6^. Searches using the website of MoHP of Nepal, PubMed, the WHO, the worldometer official website, and Google were conducted to gather the information on the number of deaths, cured, and confirmed cases of COVID-19 and reports describing the approach taken by the government to contain COVID-19 in Nepal. The search terms included “COVID-19 in Nepal” and “Prevention and management of COVID-19 in Nepal.” Data used in this article were extracted from relevant documents and websites. The figures were constructed by using Origin 2016 and GIS 10.4.1. We did not consult any databases that are privately owned or inaccessible to the public.

## Epidemic Status of COVID-19 in Nepal

The MoHP of Nepal confirmed the first and second cases of COVID-19, respectively, in January and March, in an interval of 2 months^1^ (9). As of January 22, 2021, 268,948 COVID-19 positive cases were reported, with 263,546 recovered, and 1,986 death cases^6^. This data showed nearly 0.74% death and about 98% recovery rate in Nepal. The case fatality rate (CFR) was 0.5% up to March 30 in Nepal (9). The CFR in the USA, Brazil, and Russia is similar (~2%), whereas in the South Asian Association of Regional Cooperation (SAARC) countries, the CFR varied from ~0.09 to ~4.7 % (Table 1). In total, 2,035,301 qRT-PCR tests were performed in Nepal, indicating about 13.47% current prevalence of COVID-19 among the qRT-PCR tested population as compared with 2.5% as of March 31, 2020^2^. As of reviewing, the prevalence of COVID-19 among the qRT-PCR tested population is higher than the neighboring countries, China (~0.055%) and India (~0.099%) (Table 1). In addition, up to the third quarter of 2020, <1% of the confirmed COVID-19 cases were symptomatic across all age groups, while the proportion of symptomatic cases progressively increased beyond 55 years of age from 1.3 to 9%^7^^,^^8^. Unlike Nepal, higher symptomatic cases were reported from other parts of the world during the same period (10). Understandably, the scenario of the proportion of symptomatic to asymptomatic cases remains to vary between countries and care facilities. Few possible reasons for low symptomatic cases reported in the Nepalese population may be poor health-seeking behavior and utilization of tertiary health care services (11) for mild symptomatic cases, home isolation without a diagnosis, and a high rate of self-medication practices (12).

**Table 1:** Prevalence and case fatality ratio (CFR) of COVID-19 of top leading countries, neighbor countries of Nepal, and SAARC as of Jan 28, 2021.

**Countries**	**Prevalence ratio on tested population (%)**	**Case fatality ratio (%)**
**TOP LEADING COUNTRIES**		
USA	8.596	2.687
Brazil	31.681	2.721
Russia	3.767	2.170
**NEIGHBOR COUNTRIES**		
India^*^	0.099	1.460
China	0.055	5.297
**SAARC**		
Nepal	13.129	0.755
Pakistan	6.910	2.275
Bangladesh	14.769	1.661
Bhutan	0.188	0.128
Sri Lanka	3.710	0.542
Maldives	3.867	0.099
Afghanistan	22.281	4.799

* *Belong to SAARC and top leading countries*.

**Figure 1:**
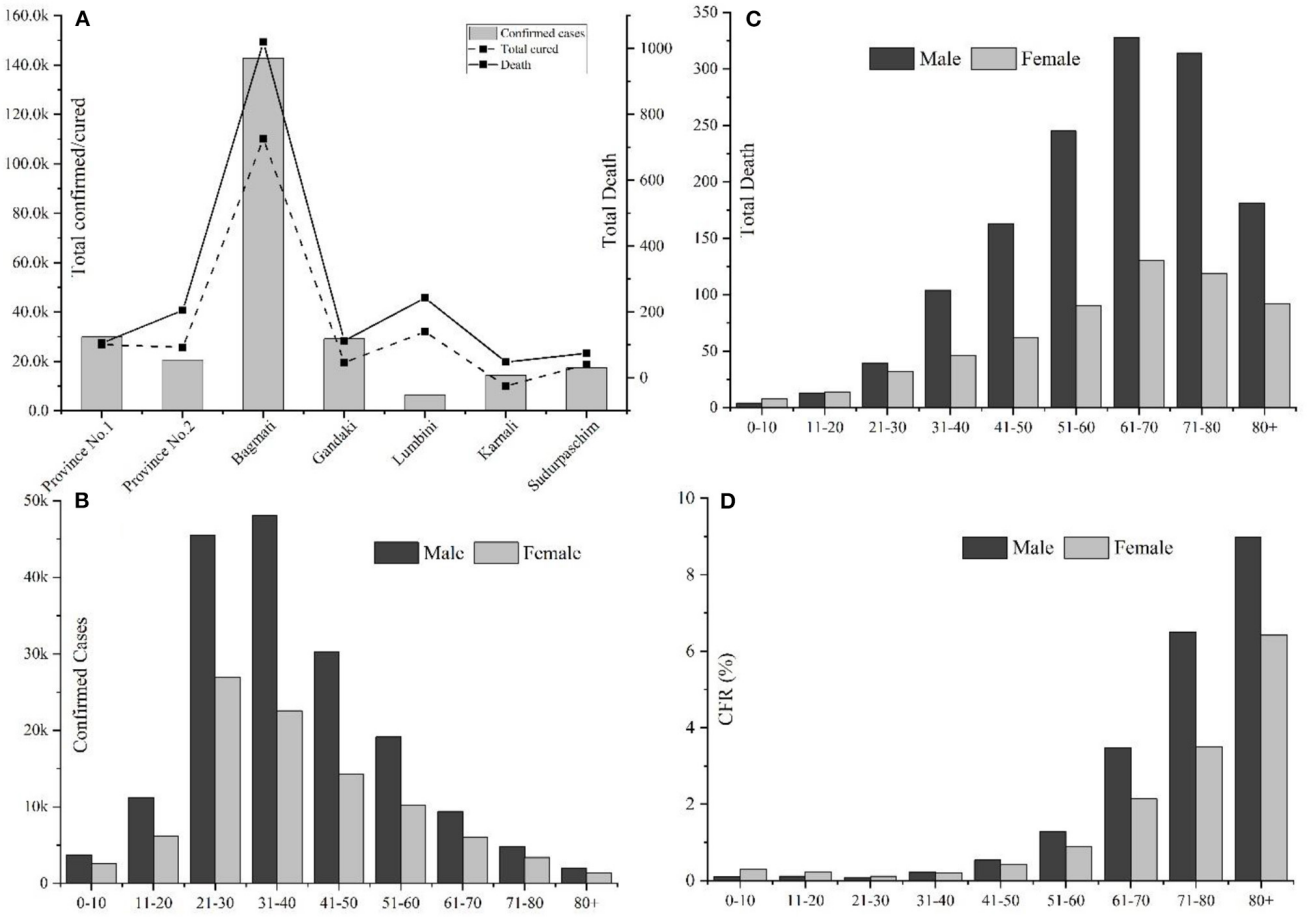
Overview of COVID-19 cases in Nepal up to January 22, 2021. **(A)** Province-wise distribution of total confirmed cases, recovery, and deaths; **(B)** Gender, age-wise distribution of COVID-19 confirmed cases; **(C)** Gender-age wise distribution of COVID-19 death cases; and **(D)** Age and gender-wise case fatality rate (CFR) in Nepal.

Among the provinces, Bagmati province (*n* = 144,278) has the highest number of confirmed cases in Nepal, followed by province no. 1 (*n* = 30,422) and Lumbini (*n* = 30,308) (Figure 1A). As depicted in Table 2, the confirmed cases of COVID-19 are distributed throughout the country in all the administrative districts. The total number of confirmed cases is highest in the Kathmandu district (*n* = 103,523) followed by Lalitpur (*n* = 16,106), Morang (*n* = 13,236), and Rupandehi (*n* = 9,708) districts and lowest in Manang (*n* = 20), Mugu (*n* = 37), Mustang (*n* = 43), and Humla (*n* = 44) districts (Table 2).

**Table 2:** District wise distribution of confirmed cases, recoveries, and deaths due to COVID-19 and total population in Nepal.

**S.N**.	**District**	**Confirmed^#^**	**Cured^#^**	**Death^#^**	Total Population^*^	**S.N**.	**District**	**Confirmed^#^**	**Cured^#^**	**Death^#^**	Total Population^*^
1	Kathmandu	103,523	100,584	738	174,4240	40	Lamjung	1,142	1,127	2	167724
2	Lalitpur	16,106	15,806	69	468,132	41	Bajhang	1,115	1,079	12	195159
3	Morang	13,236	13,166	58	965,370	42	Pyuthan	1,103	1,076	15	228102
4	Rupandehi	9,708	9,496	93	880,196	43	Arghakhanchi	1,049	1,023	4	197632
5	Bhaktapur	9,245	9,139	60	304,651	44	Sindhupalchok	1,038	1,010	16	287798
6	Sunsari	9,145	9,111	26	763,487	45	Dolakha	802	772	10	186557
7	Chitawan	8,065	7,953	68	579,984	46	Dadeldhura	793	776	2	142094
8	Kaski	7,668	7,544	20	492,098	47	Baitadi	735	706	3	250898
9	Kailali	6,111	6,036	34	775,709	48	Bajura	733	724	5	134912
10	Banke	5,123	4,960	45	491,313	49	Udayapur	682	669	2	317532
11	Jhapa	5,033	4,993	23	812,650	50	Salyan	665	656	5	242444
12	Makawanpur	4,348	4,290	42	420,477	51	Sindhuli	660	631	4	296192
13	Dang	4,189	4,119	6	552,583	52	Ramechhap	590	567	19	202646
14	Kabhrepalanchok	3,642	3,584	33	381,937	53	Parbat	546	489	28	146590
15	Parsa	3,513	3,449	50	601,017	54	Ilam	495	482	5	290254
16	Dhanusha	3,131	3,097	29	754,777	55	Jumla	475	454	2	108921
17	Nawalpur	3,100	3,068	20	310,864	56	Darchula	426	409	15	133274
18	Sarlahi	2,861	2,841	18	769,729	57	Sankhuwasabha	399	392	3	158742
19	Rautahat	2,819	2,785	30	686,722	58	Rolpa	341	319	4	224506
20	Surkhet	2,514	2,492	13	350,804	59	Myagdi	302	278	2	113641
21	Kapilbastu	2,314	2,262	14	571,936	60	Kalikot	295	292	3	136948
22	Siraha	2,294	2,270	20	637,328	61	Okhaldhunga	279	272	3	147984
23	Palpa	2,246	2,214	2	261,180	62	Dhankuta	278	269	5	163412
24	Saptari	2,187	2,141	13	639,284	63	Rukum West	267	255	12	154,272
25	Bara	2,182	2,153	21	687,708	64	Rasuwa	238	227	1	43300
26	Mahottari	2,073	2,041	29	627,580	65	Khotang	224	220	0	206312
27	Bardiya	2,004	1,927	50	426,576	66	Solukhumbu	207	204	2	105886
28	Kanchanpur	1,948	1,929	4	451,248	67	Panchthar	192	179	8	191817
29	Tanahu	1,868	1,835	13	323,288	68	Bhojpur	166	163	3	182459
30	Dhading	1,762	1,742	8	336,067	69	Terhathum	164	155	1	101577
31	Achham	1,761	1,754	4	257,477	70	Taplejung	161	160	0	127461
32	Dailekh	1,666	1,655	6	261,770	71	Jajarkot	123	119	1	171304
33	Parasi	1,642	1,596	40	332,644	72	Rukum East	114	105	3	53184
34	Gorkha	1,550	1,490	30	271,061	73	Dolpa	60	55	2	36700
35	Nuwakot	1,530	1,456	17	277,471	74	Humla	44	37	4	50858
36	Doti	1,448	1,444	1	211,746	75	Mustang	43	42	0	13452
37	Baglung	1,275	1,241	7	268,613	76	Mugu	37	35	2	55286
38	Gulmi	1,244	1,208	12	280,160	77	Manang	20	15	1	6538
39	Syangja	1,201	1,184	10	289,148	**Total**		**268948**	**263546**	**1986**	26494504

*Source:*

* *National Population Census, 2011*;

# *Ministry of Health and Population. COVID-19 Update. Available from: https://covid19.mohp.gov.np/ (Accessed on January 23, 2021)*.

Among 268,948 confirmed cases, 174,193 were males, and 94,755 were females, with a male-to-female sex ratio of 1.85. The largest number of infected cases was reported in the age group 21–30 years (26.92%, *n* = 72,396), followed by the age group of 31–40 years (26.26%, *n* = 70,648) (Figure 1B); however, the number of death cases was higher in the age group 61–70 (23%, *n* = 458) (Figure 1C). A higher death trend in old age is also observed in Europe, America, and Asian countries (13, 14). Overall, male death was ~2.33 times the death rate of females. Reports have indicated that men are at greater risk of around two time of acquiring severe outcomes of COVID-19, including hospitalizations, intensive care unit (ICU) admissions, and deaths (15). The enhanced susceptibility of males for COVID-19 associated adverse events may be correlated with the hormonal and immunological differences between males and females (15, 16). Among a total of 1,986 fatal cases (Male: *n* = 1,391; female: *n* = 595), over half (*n* = 1,166) were observed in senior adults (≥60 years). One early study among the Nepalese children suggested that male children were more commonly infected than female children (17).

**Figure 2:**
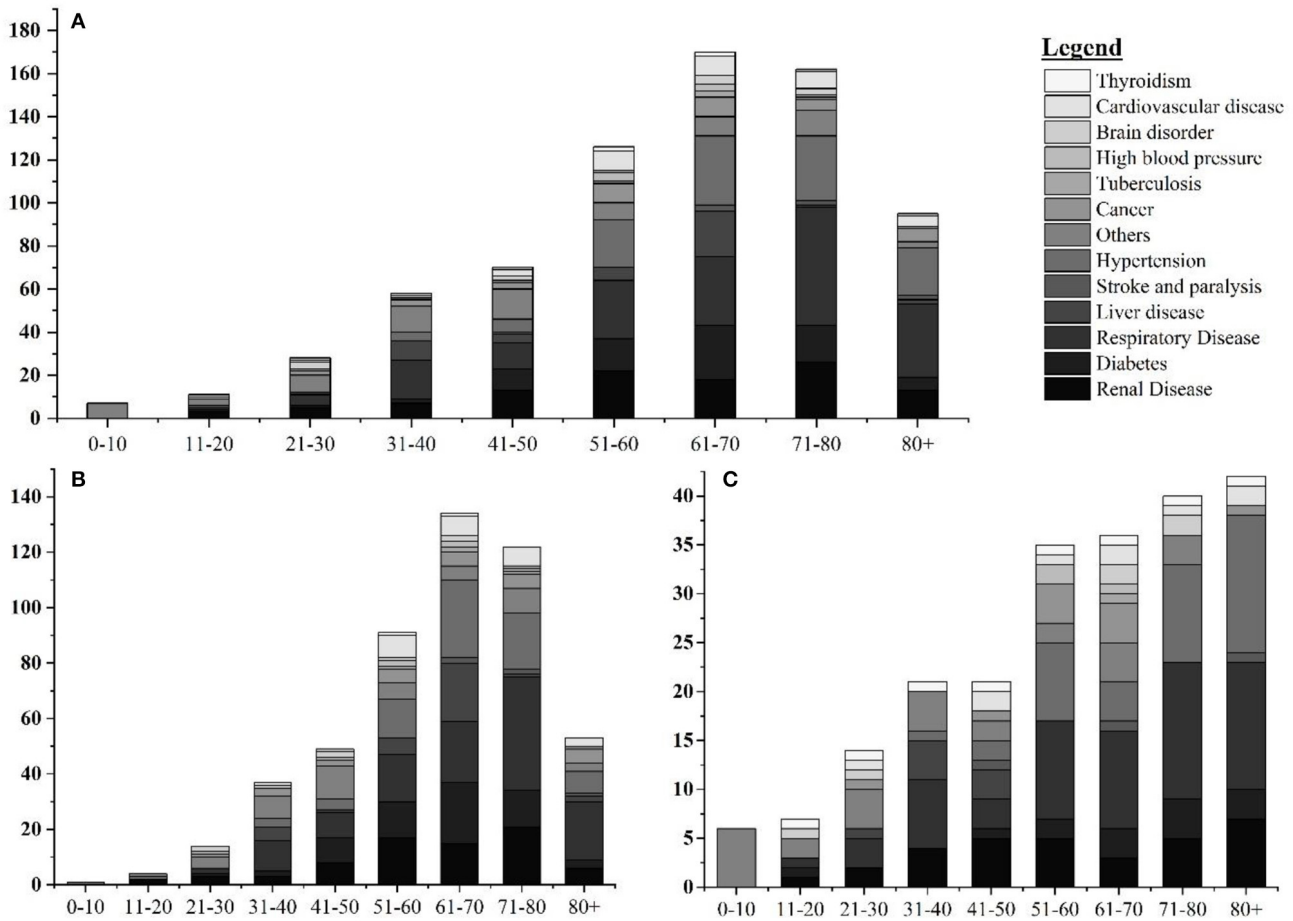
Age and gender-wise distribution fatal cases with single comorbidities. **(A)** Age-wise distribution of leading single comorbidities among COVID-19 deaths; **(B)** age-wise distribution of leading single comorbidities among COVID-19 deaths in Nepal in male; and **(C)** age-wise distribution of leading single comorbidities among COVID-19 deaths in Nepal in female.

**Figure 3:**
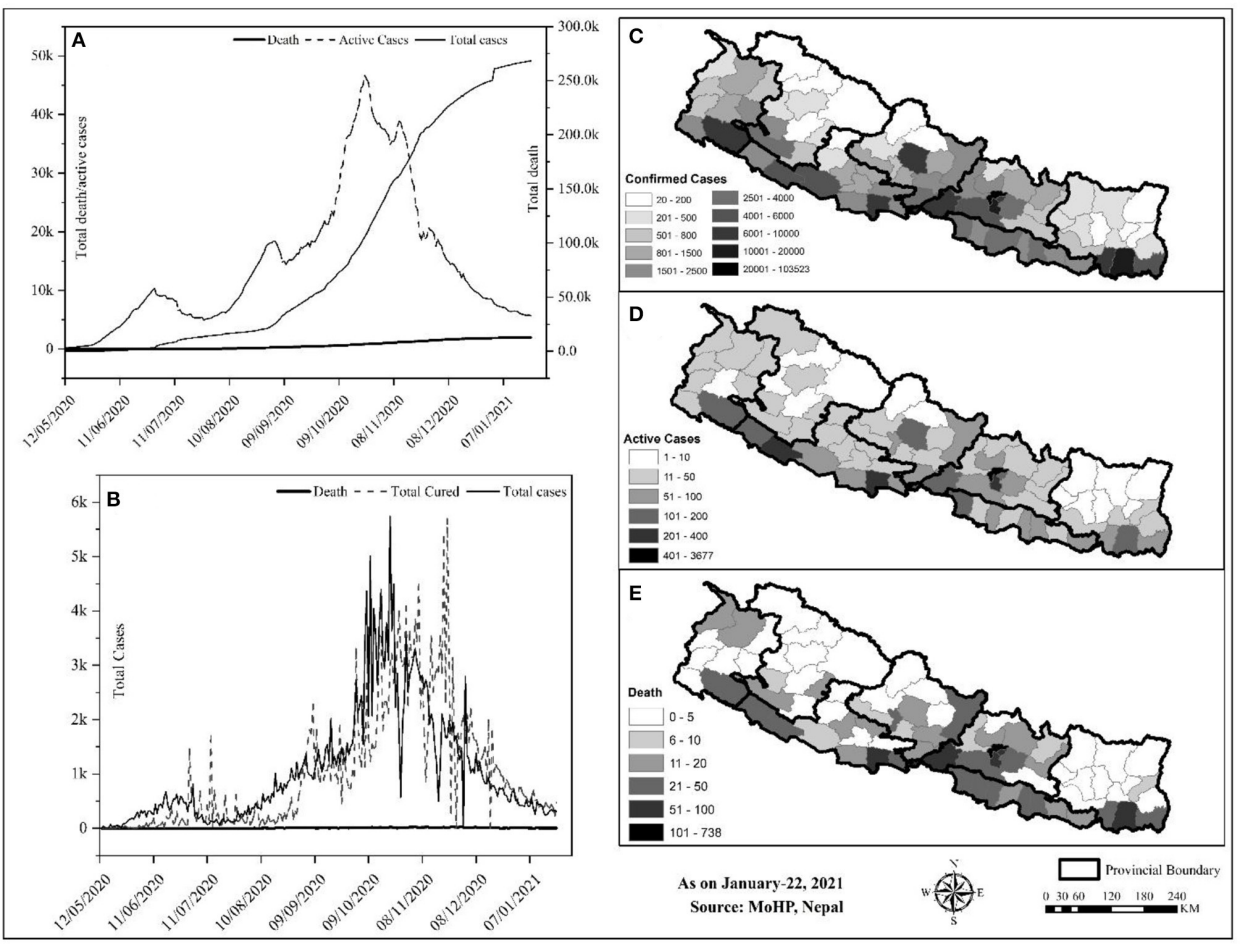
Trend and spatial distribution of COVID-19 cases in Nepal. **(A)** Cumulative trend analysis of COVID-19 cases, **(B)** daily case wise trend analysis of COVID-19, **(C–E)** spatial distribution of infected, recovered, and death cases.

Among 1,986 fatal cases (mean age: 66.15 years), 623 (31.37%), 721 (36.30%), and 642 (32.32%) were with no report of comorbidities, with single comorbidities, and with multiple comorbidities, respectively. In cases with single comorbidities, the highest incidence was reported in respiratory disease (*n* = 184) followed by hypertension (*n* = 117), renal disease (*n* = 107), diabetes (*n* = 77), liver disease (*n* = 44), and cardiovascular disease (*n* = 36) (Figure 2). Similar results are reported from other parts of the world (18). The detailed epidemiological trend analysis of COVID-19 in Nepal is shown in Figure 3.

Geographically, Nepal is divided into three distinct ecological zones, mountain, hilly, and low-plain land from north to south. Politically, Nepal is divided into 7 provinces, 77 districts, and 753 local bodies. There were multiple peaks of active cases of COVID-19 in Nepal: active cases rapidly increased from early May to early July 2020, then increased slowly up to late July and increased at a higher rate again up to the end of December, and then decreased sharply (Figure 3A). The spatial distribution of COVID-19 confirmed cases, recovery, and deaths were compared (Figures 3B–D). Approximately, 64.84% of the total confirmed cases were reported from the hill regions, with single megacity Kathmandu contributing nearly half, 33.31% of lowland-plain areas, and 1.85% of Himalayan regions. The reported cases in the megacities are relatively higher than in the other regions. The higher number of cases in megacities may be correlated with dense populations in these areas (8). In the earlier months, the testing facilities and contact tracing were limited only to few districts, including the capital, Kathmandu, which gradually became available in other parts of the country. However, the testing frequency and testing facilities are still not homogeneous due to the lack of required technical resources and professional workforces (19)^9^.

## The Response of Nepal Government to COVID-19

Nepal has adopted many readiness and response-related initiatives at the federal, provincial, and local government levels to fight against COVID-19. Initially, the government had set health desks and allocated spaces for quarantine purposes at the international airport and at the borders, crossing points of entry (PoE) with India and China^10^, to withstand the influx of many possible infected individuals from India and other countries. The open border and the politico-religious relationship with India and migrant workers returning from the Middle East, and other countries were a source of rapid transmission to Nepal^10^,^11^. The Nepal-China official border crossing points have remained closed since January 21, 2020. On March 24, 2020, the GoN imposed a complete “lockdown” of the country up to July 21, 2020. As part of the lockdown, businesses were closed, the restriction was imposed on movement within the country, workplaces were closed, travel was banned, and air transportation was halted^11^,^12^. In addition, for COVID-19 preparedness and response, the GoN developed a quarantine procedure and issued an international travel advisory notice. Closing the border was critical as Nepal and India share open borders across which citizens travel freely for business and work.

The GoN underestimated both the short and long-term impacts of border closure^11^. Around 2.8 million Nepali migrant workers work in India. Though the GoN discussed holding these workers in India with its Indian counterpart^13^, this plan did not materialize. Nepal has 1,690 km-long open borders with India, which could not keep migrant workers long despite the restrictions implemented by both governments^12^. As a consequence, the majority of COVID-19 cases were in the districts along the Indo-Nepal border. The decision of the government to lockdown the country from March 10, 2020, without sufficient preparation pushed daily wage laborers in urban areas to lose their jobs, and, hence, they were trapped without food or money. Ultimately, after a couple of days of lockdown, both migrant workers and daily wage laborers started walking the long way home due to the economic crisis.

As per the cabinet decision on March 25, 2020, Nepal established a COVID-19 response fund, developed a relief package^13^, and distributed relief to families in need through a “one door policy”^13^ designed to reduce the COVID-19 impact; however, there were several gaps: the selection of families was unfair, GoN delayed the procurement of relief, relief packages did not include cash, and relief materials were inadequate and substandard ^14^^,^^15^. The government has not adequately taken into account the impact of COVID-19 on the socio-economic sector. For instance, people participated in meetings, rallies, political demonstrations, and protests, where the virus could quickly spread among a large group of people. The government has, yet, to develop a stimulus package for social and economic recovery at the micro and macro levels. As the government has allocated $788 million for the health sector for the fiscal year (July–June 2020), a budget of 32% larger than the previous fiscal year, it should address the COVID-19 impact on the socio-economic front^16^. There is an opportunity to integrate all fragmented social protection schemes to strengthen socio-economic conditions and to emphasize more tremendous efforts, capacities, and resources to cope with the likely impacts of the COVID-19 pandemic^16^.

In addition, a minimal standard of quarantine as per the “Quarantine Operation and Management Protocol” (2076 B.S.) and “Standards for Home Quarantine” were imposed for all provinces^16^^,^^17^. The Sukraraj Infectious and Tropical Disease Hospital (SITDH) in Teku, Kathmandu, was designated by GoN as the primary hospital for COVID-19 cases along with Patan Hospital, the Armed Police Forces Hospital, in the Kathmandu Valley, followed by twenty-four hubs, and four satellite hospitals across the country^18^. Similarly, MoHP updated the National Public Health Laboratory (NPHL) capacity for confirmatory laboratory diagnosis of the COVID-19 from January 27, 2020, followed by the regional laboratory. The interim guideline for the establishing and operating of molecular laboratories for COVID-19 testing in Nepal was imposed to make uniformity in the test results^14^. Furthermore, the NPHL organized the training of trainers for laboratory staff in collaboration with the Medical Laboratory Association of Nepal^19^. Ministry of Health and Population established two hotline numbers (1115 and 1133) to address public concerns, and prepared and disseminated regular press briefings, and improved its websites to channel appropriate information to the public. Besides, MoHP also conveyed decisions, notices, and situation updates periodically through its websites. Further, the Health Emergency Operation Centre (HEOC) of MoHP launched a “Viber communication group” to circulate updates on COVID-19^11, 13^. Early testing and timely contact tracing are crucial restrictive policies to control the spreading of the SARS-CoV-2 virus (20, 21); however, in the earlier days of the pandemic, Nepal could not perform enough diagnostic tests and timely contact tracing; it resulted in a crucial time lag in identifying and isolating COVID-19 patients and caused delays in the ability of government to respond to the pandemic adequately. To alert and improve the testing and tracing response of the government, youth-led protests were carried out in different parts of the country^20^. Health Sector Emergency Response Plan was implemented in May 2020, focusing on the COVID-19 pandemic. This plan intends to prepare and strengthen the health system response capable of minimizing the adverse impact of the COVID-19 pandemic. Government of Nepal devised a comprehensive plan on March 27, 2020, for quarantining people who arrived in Nepal from COVID-19 affected countries. The GoN had initially airlifted 175 Nepalese from six cities across Hubei Province of China on February 15, 2020, followed by Middle East countries, Australia, and so on^13^.

Ministry of Health and Population engaged in developing, endorsing, improving, and disseminating contextualized technical guidelines, standard operating procedures (SOPs), tools, and training in all other critical aspects of the response to COVID-19, for instance, surveillance, case investigation, laboratory testing, contact tracing, case detection, isolation and management, infection prevention and control, empowering health and community volunteers, media communication and community engagement, rational use of personal protective equipment (PPE), requirements of drugs and equipment for case management and public health interventions, and continuity of essentials services^13^ (15). The major contextualized technical guidelines, SOPs, tools, and training materials developed by GoN to respond to COVID-19^21^^,^^22^^,^^23^^,^^24^^,^^25^^,^^26^^,^^27^^,^^28^^,^^29^^,^^30^ were listed in Table 3.

**Table 3:** Major contextualized technical guidelines, standard operating protocols, tools, and training materials developed by the Government of Nepal (GoN) to respond to COVID-19.

**S.No**.	**Date**	**Major contextualized technical guidelines, SOPs, tools, and training materials**
1	April, 2020	Guidelines for the management of front-line healthcare service providers and other workers involved in the management of COVID-19 cases
2	April, 2020	Interim guidance for the operation of nutrition rehabilitation in the context of COVID-19
3	April, 2020	Interim pocketbook of clinical management of COVID-19 in the healthcare setting and Infection Prevention and Control pocket booklet; interim guideline for the establishment and operationalization of molecular laboratory for COVID-19 testing in Nepal; a guideline on safety measures to be taken at the point of entry
4	May, 2020	Standard operating procedure of cleaning and decontamination of the ambulance used in COVID-19. The Department of Ayurveda and alternative medicine has recently published national guidelines on preventive measures and management protocol for COVID-19 in Nepal
5	May, 2020	Guidelines for the management of dead bodies of people who died from COVID-19, COVID-19 cases isolation management, and COVID-19 emergency medical teams (EMDT) mobilization
6	June, 2020	MoHP has issued a guideline on minimum standards for donor agencies/partner organizations for COVID-19 logistics support to the MoHP-2020
7	July, 2020	Guidance on testing of high-risk groups and random testing of people in communities at Kathmandu Valley including other high-risk COVID-19 affected districts to detect community transmission
8	August, 2020	Standards for the service delivery of senior citizens in the context of COVID-19

Ministry of Health and Population and supporting organizations, such as United Nations Development Program (UNDP), UNICEF, and World Vision managed crucial supplies of PPE, facemasks, gloves, and sanitizers to ensure the protection of frontline workers and supporting staffs^13^^,^^30^^,^^31^^,^^32^. The frontline media of the nation increased online awareness programs *via* the involvement of celebrities, doctors, and experts of microbiology and infectious diseases on physical distancing and the importance and use of masks and sanitizers to prevent the COVID-19 contagion. In addition, camping programs were launched by the involvement of youth volunteers of the community in central Nepal^33^.

Government of Nepal received funds from the World Bank ($29 million), the United States of America ($1.8 million), and Germany ($1.22 million) to keep people protected from COVID-19 through health systems preparedness, emergency response, and research. In addition, support from UNICEF and countries, including China, India, and the USA, in the form of emergency medical supplies and equipment were received within January 2020 to March 2020. Private companies, corporate houses, business organizations, and individuals have also contributed to the prevention, control, and treatment fund of coronavirus ($13.8 million), established by GoN to cope with COVID-19. The Prime Minister Relief Fund is also expected to be utilized. The GoN allowed international NGOs to divert 20% of their program budget to COVID-19 preparedness and response; for instance, the Social Welfare Council has allocated $226 million^31^^,^^33^^,^^34^^,^^35^^,^^36^^,^^37^.

The GoN has formed a committee to coordinate the preparedness and response efforts, including the MoHP, Ministry of Home Affairs, Ministry of Foreign Affairs, Ministry of Finance, Ministry of Culture, Tourism and Civil Aviation, Ministry of Urban Development, Nepal Army, Nepal Police, and Armed Police Force. The Humanitarian Country Team (HCT) includes the Red Cross Movement and civil society organizations (national and international NGOs). Under the joint leadership of the office of Resident Coordinator and the WHO, the HCT has initiated contingency planning and preparedness interventions, including the dissemination of communications materials to raise community-level awareness across the country^21^. The clusters led by the GoN and co-led by the International Astronomical Search Collaboration (IASC) cluster leads and partners are working on finalizing contingency plans, which will be consolidated into an overall joint approach with the Government and its international partners. The UN activated the provincial focal point agency system to support coordination between the international community and the GoN at the provincial level^21^.

However, despite these robust efforts implemented by GoN, few lapses existed. Examples are the following: issues of inconsistent implementation of immigration policies usually at Indo-Nepal borders^38^^,^^39^^,^^40^, shortage and misuse of crucial protective suits and other supplies in hospitals, the ease and the end of lockdown, lack of poor infrastructure facilities, and continuous spread of COVID-19 across the country (19). The GoN decided to lift the lockdown effective from July 22, 2020, completely; however, the socio-administrative and health measures with the potential for high-intensity transmission (colleges, seminars, training, workshops, cinema halls, party palaces, dance bars, swimming pools, religious places, etc.) remained closed until the following directive as of September 1, 2020. Long route bus services and domestic and international passenger flights were halted until August 1, 2020^41^. A high-level committee at the MoHP has requested all satellite hospitals (public, private, and others) to allocate 20% of their beds for COVID-19 cases. The respective hub hospitals coordinate with the HEOC and satellite hospitals to manage COVID-19 cases^42^. After lifting lockdown for 3 weeks, the federal government has given authority to local administrations to decide on restrictions and lockdown measures as COVID-19 cases continue to rise. In addition, the authority to impose necessary restrictions if COVID-19 active cases surpass the threshold of 200 was given to the Chief District Officer (CDO)^43^. Since March 2020, all the central hospitals, provincial hospitals, medical colleges, academic institutions, and hub-hospitals were designated to provide treatment care for COVID-19 cases. At this stage of operation, the major challenges for the COVID-19 response were managing quarantine facilities, lack of enough human resources, having limited laboratories for testing, and availability of limited stock of medical supplies, including PPEs^14^. To the best of our knowledge, this pandemic is the most extensive public health emergency the GoN faced in its recent history.

There is no doubt that GoN has taken major initiatives to fight the COVID-19 pandemic. The MoHP, together with associated national and international organizations are closely monitoring and evaluating the signs of outbreaks, challenges, and enforcing the plan and strategies to mitigate the possible impact; however, many challenges and difficulties, such as management of testing, hospital beds, and ventilators, quarantine centers, frontline staffs, movement of people during the lockdown, are yet to be solved^18^^,^^30^^,^^38^,^44^^,^^45^^,^^46^^,^^47^. Therefore, in the opinion of the authors, we recommend some steps to be implemented as soon as possible to mitigate and lessen the impacts of COVID-19 (Table 4).

**Table 4:** Major steps taken by GoN and way forward in the response to COVID-19 outbreak.

**S. N**.	**Date**	**Key steps taken by the GoN**	**Way ahead for Nepal**
1	January, 2020	Early warning and reporting system (EWARS)—daily and weekly bulletin: Nepal	Increase the tracing and testing
2	March, 2020	Formed a high-level coordination committee led by the Deputy Prime Minister	Impact analysis and current and post-pandemic recovery plans and strategies
3	March, 2020	Non-pharmacological interventions such as lockdown, social distancing, quarantine, travel restrictions, media awareness	Capacity building training for healthcare personal and front-line workers
4	March, 2020	Postponed less essential scheduled campaigns program, for instance, Visit Nepal 2020	Training and monitoring for vaccination
5	March, 2020	Discussion with experts to outline new strategies, frame action plans and implement interventions	
6	April, 2020	Collaboration, networking, and coordination with ministries, global health sectors, NGOs, and INGOs. Instructed INGOs to divert up to 20% of their program budget to tackle COVID-19	Preparation of a robust database and information system
7	April, 2020	Guidelines for SOP/Tools/Protocol for management of COVID-19	
8	April, 2020	Establishment of grain banks for needy families	
9	May, 2020	Acceptance of donation fund and set up an emergency COVID-19 fund at the federal level, province level, and local level	Strengthening of laboratory and hospital facilities, and motivation for frontline staffs
10	May, 2020	Significantly strengthened ICU, ventilators, laboratory facilities, expansion of laboratory and testing	Systematization of quarantine centers and isolation beds
11	August, 2020	Procurement and supplies of PPE and Coronavirus Insurance Program	Volunteer mobilization and increase awareness and knowledge for citizens

To strengthen its coordination mechanism, the government formed a team to monitor conditions and measures applied to control the outbreak; a COVID-19 coordination committee^11^ to coordinate the overall response, and a COVID-19 crisis management center^14^ to coordinate daily operations; however, these teams and committees did not function efficiently because roles and authorities were not delegated to ministries and government. A new institution was created, instead of using the National Disaster Risk Reduction and Management Authority (NDRRMA)^48^, which enhanced additional confusion. The MoHP is responsible for overall policy formulation, planning, organization, and coordination of the health sector at federal, provincial, district, and community levels during the COVID-19 pandemic situation. Allegedly, there is an opportunity to strengthen coordination among the tiers of governments by following protocols and guidance for effective preparedness and response. For example, some quarantine centers were so poorly run that, in turn, could potentially develop into breeding grounds for the COVID-19 transmission^15^.

Finally, this study only focuses on analyzing COVID-19 data extracted from the MoHP database for 1 year. Furthermore, we did not quantify the effectiveness of the strategies of GoN and the role of non-governmental organizations and authorities to combat COVID-19 in Nepal.

## Conclusion

This study provides an insight into the impacts of the COVID-19 pandemic from the Nepalese context for the period of first-wave from January 2020 to January 2021. Despite the several initiatives taken by the GoN, the current scenario of COVID-19 in Nepal is yet to be controlled in terms of infections and mortality. A total of 268,948 confirmed cases and 1,986 deaths were reported in one year period. The maximum number of cases were reported from Bagmati province (*n* = 144,278), all of the 77 districts were affected. The cases showing highly COVID-specific symptoms were low (<1%) in comparison with the reports across the globe (10), which may be because the average age of the Nepalese population is younger than many of the highly affected European countries. The other reasons may be differences in demographic characteristics, sampling bias, healthcare coverage, testing availability, and inconsistencies relating to the reporting of the data included in the current study. Both the number of infections and deaths are higher in males than in females. Despite the age, testing and positivity, hospital capacity and hospital admission criterion, demographics, and HDI index, the overall case fatality was reported to be less than in some other developed countries (Table 1). Consistent with reports from other countries (22, 23), the death rate is higher in the old age group (Figure 1). Spatial distribution displayed the cases, which are majorly distributed in megacities compared with the other regions of the country.

Based on this assessment, in addition to the WHO COVID-19 infection prevention and control guidance^49^, some recommendations, such as massive contact tracing, improving bed capacity in health care settings and rapid test, proper management of isolation and quarantine facilities, and advocacy for vaccines, may be helpful for planning strategies and address the gaps to combat against the COVID-19. Notably, the recommendations provided could benefit the governmental bodies and concerned authorities to take the appropriate decisions and comprehensively assess the further spread of the virus and effective public health measures in the different provinces and districts in Nepal. In this review, we have summarized the ongoing experiences in reducing the spread of COVID-19 in Nepal. The Nepalese response is characterized by nationwide lockdown, social distancing, rapid response, a multi-sectoral approach in testing and tracing, and supported by a public health response. Overall, the broader applicability of these experiences is subject to combat the COVID-19 impacts in different socio-political environments within and across the country in the days to come.

## Author Contributions

BB: Conceptualization, writing, and original draft preparation. KB, BB, and AG: data curation. BB, RP, TB, SD, NP, and DG: writing, review, and editing. All authors contributed to the article and approved the submitted version.

## Conflict of Interest

KB and AG were employed by Nepal Environment and Development Consultant Pvt. Ltd., in Kathmandu, Nepal. The remaining authors declare that the research was conducted in the absence of any commercial or financial relationships that could be construed as a potential conflict of interest.

## Footnotes

^1^Worldometer. *COVID-19 Coronavirus Pandemic*. (2020). Available online at: https://www.worldometers.info/coronavirus/ (accessed January 15, 2021).

^2^Worldometer. *Nepal Population*. (2020). Available online at: https://www.worldometers.info/world-population/nepal-population/ (accessed January 15, 2021).

^3^Trading Economics. *Nepal GDP*. (2020). Available online at: https://tradingeconomics.com/nepal/gdp (accessed January 15, 2021).

^4^UNDP. *Human Development Reports*. (2020). Available online at: http://hdr.undp.org/en/countries/profiles/NPL (accessed January 15, 2021).

^5^World Health Organization. *COVID-19 Nepal: Preparedness and Response Plan (NPRP)*. (2020). Available online at: https://www.who.int/docs/default-source/nepal-documents/novel-coronavirus/covid-19-nepal-preparedness-and-response-plan-(nprp)-draft-april-9.pdf?sfvrsn=808a970a_2 (accessed January 15, 2021).

^6^Ministry of Health and Population. *COVID-19 Update*. (2020). Available online at: https://covid19.mohp.gov.np/ (accessed January 15, 2021).

^7^World Health Organization. *WHO Nepal Situation Updates-19 on COVID-19, 2020*. (2020). Available online at: https://www.who.int/docs/default-source/nepal-documents/novel-coronavirus/who-nepal-sitrep/19-who-nepal-sitrep-covid-19.pdf?sfvrsn=c9fe7309_2 (accessed January 15, 2021).

^8^World Health Organization. *WHO Nepal Situation Updates-22 on COVID-19, 2020*. (2020). Available online at: https://www.who.int/docs/default-source/nepal-documents/novel-coronavirus/who-nepal-sitrep/22-who-nepal-sitrep-covid-19.pdf?sfvrsn=df7c946a_2 (accessed January 15, 2021).

^9^World Health Organization. (2020). *WHO Nepal Situation Updates-16 on COVID-19, 2020*. (2020). Available online at: https://www.who.int/docs/default-source/nepal-documents/novel-coronavirus/who-nepal-sitrep/16–who-nepal–sitrep-covid-19-07082020-final.pdf?sfvrsn=53c5360f_2 (accessed January 15, 2021).

^10^Bhattarai, KD. *South Asian Voices: COVID-19 and Nepal's Migration Crisis*. Available online at: https://southasianvoices.org/covid-19-and-nepals-migration-crisis/ (accessed January 15, 2021).

^11^*GRADA WORLD Nepal: Government announces nationwide lockdown from March 24–31/update*. Available online at: https://www.garda.com/crisis24/news-alerts/326601/nepal-government-announces-nationwide-lockdown-from-march-24-31-update-4 (accessed January 15, 2021).

^12^Gautam D. *NDRC. Nepal's Readiness and Response to COVID-19*. (2020). Available online at: https://www.preventionweb.net/files/71274_71274nepalsreadinessandresponsetopa.pdf (accessed January 15, 2021).

^13^*Building Back Better (BBB) from COVID-19: World Vision Policy Brief on Building Back Better from COVID-19*. (2020). Available online at: https://www.wvi.org/sites/default/files/202005/World%20Vision%20Policy%20Brief%20on%20Building%20Back%20Better_25%20May%202020.pdf (accessed January 15, 2021).

^14^World Health Organization. *WHO Nepal Situation Updates-1 on COVID-19, 2020*. (2020). Available online at: https://www.who.int/docs/default-source/nepal-documents/novel-coronavirus/who-nepal-sitrep/who-nepal–sitrep-covid-19-20apr2020.pdf?sfvrsn=c788bf96_2 (accessed January 15, 2021).

^15^GoN MoHP. *Health Sector Emergency Response Plan COVID-19 Pandemic 2020*. (2020). Available online at: https://www.who.int/docs/default-source/nepal-documents/novel-coronavirus/health-sector-emergency-response-plan-covid-19-endorsed-may-2020.pdf?sfvrsn=ef831f44_2 (accessed January 15, 2021).

^16^Gautam, D. *The COVID-19 Crisis in Nepal: Coping Crackdown Challenges. National Disaster Risk Reduction Centre, Kathmandu, Nepal. Issue 3, 2020*. Available online at: https://www.alnap.org/help-library/the-covid-19-crisis-in-nepal-coping-crackdown-challenges (accessed January 30, 2021).

^17^Gautam, D. *Fear of COVID-19 Overshadowing Climate-Induced Disaster Risk Management*. Available online at: https://www.spotlightnepal.com/2020/05/08/fear-covid-19-overshadowing-climate-induced-disaster-risk-management/ (accessed January 30, 2021).

^18^Pradhan TR. *The Kathmandu Post. Nepal Goes Under Lockdown for a Week Starting 6am Tuesday*. Available online at: https://kathmandupost.com/national/2020/03/23/nepal-goes-under-lockdown-for-a-week-starting-6am-tuesday (accessed January 30, 2021).

^19^World Health Organization. *WHO Nepal Situation Updates-3 on COVID-19, 2020*. (2020). Available online at: https://www.who.int/docs/default-source/nepal-documents/novel-coronavirus/who-nepal-sitrep/who-nepal–situpdate-3-covid-19-06052020.pdf?sfvrsn=714d14c4_2 (accessed January 30, 2021).

^20^Jha IC. *The Rising Nepal. MoHP Sets Forth Standards for Home Quarantine*. Available online at: https://risingnepaldaily.com/featured/mohp-sets-forth-standards-for-home-quarantine (accessed January 30, 2021).

^21^The Kathmandu Post. *Youth-Led Protests Against the Government's Handling of Covid-19 Spread to Major Cities*. (2020). Available online at: https://kathmandupost.com/national/2020/06/12/youth-led-protests-against-the-government-s-handling-of-covid-19-spread-to-major-cities (accessed January 30, 2021).

^22^World Health Organization. *WHO Nepal Situation Updates-2 on COVID-19, 2020*. (2020). Available online at: https://www.who.int/docs/default-source/nepal-documents/novel-coronavirus/who-nepal-sitrep/who-nepal–sitrep-covid-19-29apr2020.pdf?sfvrsn=dac001bf_2 (accessed January 30, 2021).

^23^World Health Organization. *WHO Nepal Situation Updates-4 on COVID-19, 2020*. (2020). Available online at: https://www.who.int/docs/default-source/nepal-documents/novel-coronavirus/who-nepal-sitrep/who-nepal–situpdate-4-13052020.pdf?sfvrsn=630b68ea_6 (accessed January 30, 2021).

^24^World Health Organization. *WHO Nepal Situation Updates-18 on COVID-19, 2020*. (2020). Available online at: https://www.who.int/docs/default-source/nepal-documents/novel-coronavirus/who-nepal-sitrep/18-who-nepal-sitrep-covid-19-23082020.pdf?sfvrsn=6fb20500_2 (accessed February 5, 2021).

^25^World Health Organization. *WHO Nepal Situation Updates-5 on COVID-19, 2020*. (2020). Available online at: https://www.who.int/docs/default-source/nepal-documents/novel-coronavirus/who-nepal-sitrep/5-who-nepal–situpdate-covid-19-20052020-final.pdf?sfvrsn=7552c8ba_4 (accessed February 5, 2021).

^26^World Health Organization. *WHO Nepal Situation Updates-7 on COVID-19, 2020*. (2020). Available online at: https://www.who.int/docs/default-source/nepal-documents/novel-coronavirus/who-nepal-sitrep/7-who-nepal–situpdate-covid-19-03062020-final.pdf?sfvrsn=87f582d6_2 (accessed February 5, 2021).

^27^World Health Organization. *WHO Nepal Situation Updates-8 on COVID-19, 2020*. (2020). Available online at: https://www.who.int/docs/default-source/nepal-documents/novel-coronavirus/who-nepal-sitrep/8-who-nepal–situpdate-covid-19.pdf?sfvrsn=ce5ecb07_2 (accessed February 5, 2021).

^28^World Health Organization. *WHO Nepal Situation Updates-10 on COVID-19, 2020*. (2020). Available online at: https://www.who.int/docs/default-source/nepal-documents/novel-coronavirus/who-nepal-sitrep/10-who-nepal–situpdate-covid-19-24062020.pdf?sfvrsn=c7f99a61_8 (accessed February 5, 2021).

^29^World Health Organization. *WHO Nepal Situation Updates-13 on COVID-19, 2020*. (2020). Available online at: https://www.who.int/docs/default-source/nepal-documents/novel-coronavirus/who-nepal-sitrep/13–who-nepal–situpdate-covid-19-17072020-v4.pdf?sfvrsn=fc0f19cc_2 (accessed February 5, 2021).

^30^World Health Organization. *WHO Nepal Situation Updates-17 on COVID-19, 2020*. (2020). Available online at: https://www.who.int/docs/default-source/nepal-documents/novel-coronavirus/who-nepal-sitrep/17-who-nepal-sitrep-covid-19-15082020.pdf?sfvrsn=68a53b32_2 (accessed February 10, 2021).

^31^UN. *Nepal Information Platform, COVID-19 Nepal: Preparedness and Response Plan*. Available online at: http://un.org.np/reports/covid-19-nepal-preparedness-and-response-plan (accessed February 10, 2021).

^32^*UNICEF for Every Child, Supporting COVID-19 Readiness and Response in the West of Nepal*. Available online at: https://www.unicef.org/nepal/stories/supporting-covid-19-readiness-and-response-west-nepal (accessed February 10, 2021).

^33^UNDP. *Enhancing Public Awareness on COVID-19 Through Communications*. Available online at: https://www.np.undp.org/content/nepal/en/home/presscenter/articles/2020/Enhancing-public-awareness-of-COVID-19-through-communications.html (accessed February 10, 2021).

^34^The World Bank. *The Government of Nepal and the World Bank sign $29 Million Financing Agreement for Nepal's COVID-19 (Coronavirus) Response*. Available online at: https://www.worldbank.org/en/news/press-release/2020/04/03/world-bank-fast-tracks-29-million-for-nepal-covid-19-coronavirus-response (accessed February 10, 2021).

^35^Khatri PP. *The Rising Nepal. Govt Receives Over Rs 1.59 Bln In Anti-COVID-19 Fund*. Available online at: https://risingnepaldaily.com/main-news/govt-receives-over-rs-159-bln-in-anti-covid-19-fund (accessed February 10, 2021).

^36^Dahal A. *Govt Does U-Turn to Let NGOs Hand Out Medical Supplies, Food, Cash directly*. Available online at: https://myrepublica.nagariknetwork.com/news/govt-does-u-turn-to-let-ingos-hand-out-medical-supplies-food-cash-directly/ (accessed February 10, 2021).

^37^Rijal A. *The Rising Nepal. China Gives Anti-Corona Medical Aid*. Available online at: https://risingnepaldaily.com/main-news/china-gives-anti-corona-medical-aid (accessed February 10, 2021).

^38^Nepali Sansar. *Nepal Receives 23 Tons ‘COVID-19 Medical Equip' As Gifts from India*. (2020). Available online at: https://www.nepalisansar.com/coronavirus/nepal-receives-23-tons-covid-19-medical-equip-as-gifts-from-india/ (accessed February 10, 2021).

^39^Koirala S, Bhattarai, S. *My Republica. Protect Frontline Healthcare Workers*. Available online at: https://myrepublica.nagariknetwork.com/news/protect-frontline-healthcare-workers/ (accessed February 10, 2021).

^40^Halder R. *Lockdowns and national borders: How to manage the Nepal-India border crossing during COVID-19*. Available online at: https://blogs.lse.ac.uk/southasia/2020/05/19/lockdowns-and-national-borders-how-to-manage-the-nepal-india-border-crossing-during-covid-19/ (accessed February 10, 2021).

^41^Raturi K. *How Is Nepal Tackling COVID Crisis & Reverse Migration of Workers?* Available online at: https://www.thequint.com/voices/opinion/india-nepal-border-coronavirus-pandemic-migrant-workers-exodus-reverse-migration-unemployment (accessed February 10, 2021).

^42^World Health Organization. *WHO Nepal Situation Updates-14 on COVID-19, 2020*. (2020). Available online at: https://www.who.int/docs/default-source/nepal-documents/novel-coronavirus/who-nepal-sitrep/14–who-nepal–sitrep-covid-19-26072020.pdf?sfvrsn=65868c9e_2 (accessed February 10, 2021).

^43^World Health Organization. *WHO Nepal Situation Updates-19 on COVID-19, 2020*. (2020). Available online at: https://www.who.int/docs/default-source/nepal-documents/novel-coronavirus/who-nepal-sitrep/19-who-nepal-sitrep-covid-19.pdf?sfvrsn=c9fe7309_2 (accessed February 10, 2021).

^44^Prasain S, Pradhan TR. *The Kathmandu Post*. Available online at: https://kathmandupost.com/politics/2020/08/12/nepal-braces-for-a-return-to-locked-down-life-as-rise-in-covid-19-cases-rings-alarm-bells (accessed February 10, 2021).

^45^NHPL. *Information regarding Novel Corona Virus*. (2020). Available online at: https://www.nphl.gov.np/page/ncov-related-lab-information (accessed February 10, 2021).

^46^NHRC. *Assessment of Health-related Country Preparedness and Readiness of Nepal for Responding to COVID-19 Pandemic Preparedness and Readiness of Government of Nepal Designated COVID Hospitals*. (2020). Available online at: http://nhrc.gov.np/wp-content/uploads/2020/06/Fact-sheet-Preparedness-and-Readiness-of-Government-of-Nepal-Designated-COVID-Hospitals.pdf (accessed February 10, 2021).

^47^Koirala S. *Comprehensive response to COVID 19 in Nepal*. Available online at: https://en.setopati.com/blog/152612 (accessed February 10, 2021).

^48^*National Disaster Risk Reduction and Management Authority, Ministry of Home Affairs, Government of Nepal*. Available online at: https://covid19.ndrrma.gov.np/ (accessed February 10, 2021).

^49^World Health Organization. *Infection Prevention and Control Guidance - (COVID-19)*. (2021). Available online at: https://www.who.int/emergencies/diseases/novel-coronavirus-2019/advice-for-public (accessed February 10, 2021).
